# 囊腔型肺癌的诊疗进展

**DOI:** 10.3779/j.issn.1009-3419.2023.101.29

**Published:** 2023-10-20

**Authors:** Jinxu YANG, Ying CHEN, Yujie LEI, Yunchao HUANG

**Affiliations:** 650118 昆明，昆明医科大学第三附属医院胸外一科; Department of Thoracic Surgery I, The Third Affiliated Hospital of Kunming Medical University, Kunming 650118, China

**Keywords:** 肺肿瘤, 囊腔型肺癌, 发病机制, 早期诊断, 影像特点, 临床病理特征, 预后, Lung neoplasms, Lung cancer associated with cystic airspaces, Pathogenesis, Early diagnosis, Image characteristics, Clinicopathological features, Prognosis

## Abstract

囊腔型肺癌（lung cancer associated with cystic airspaces, LCCA）是一种影像学上表现为肿瘤内或边缘存在囊性空腔的肺癌类型。由于易被误诊或漏诊，LCCA患者的预后不良，因此需要更多临床研究来揭示其特点。目前已有4种影像学分类系统，并且近年来对LCCA的关注度逐渐升高，尤其是对其影像学特征进行了一定的研究。既往结果显示，LCCA的病理特征与影像学表现存在相关性，但对于与肺癌相关的驱动基因突变和分子分型的研究仍不充分。由于LCCA难以早期诊断，预后较一般类型肺癌差，本文综述了LCCA的定义、病因与发病机制、影像学特征与诊断依据、组织学和病理学特征及预后等方面的研究进展，以期为临床决策提供参考。

## Abstract (en)

Lung cancer associated with cystic airspaces (LCCA) is a type of lung cancer characterized by the presence of cystic cavities in or around the tumor on imaging. Due to its high potential for misdiagnosis or underdiagnosis, the prognosis of LCCA patients is poor, necessitating further large-scale clinical studies to elucidate its characteristics. Currently, four imaging classification systems exist, and there has been a progressive increase in attention towards LCCA, particularly with regard to the study of its imaging features. The results indicate a correlation between the pathological features and imaging findings of LCCA; however, research on driver gene mutations and molecular subtyping associated with lung cancer remains insufficient. Due to the challenges associated with early diagnosis and the poorer prognosis compared to general types of lung cancer, this paper comprehensively reviews the research progress on LCCA, including its definition, etiology, pathogenesis, imaging features, histological and pathological features, treatment, and prognosis, aiming to serve as a valuable resource for clinical decision-making.

肺癌在胸部电子计算机断层扫描（computed tomography, CT）上常表现为磨玻璃、部分实性和实性的结节或肿块，但有一种与囊性空腔（下称“囊腔”，cystic airspace, CA）相关的特殊肺癌类型——囊腔型肺癌（lung cancer associated with cystic airspaces, LCCA），影像学表现为在囊性空腔的内部或边缘存在实性或亚实性成分。

1941年Womack和Graham^[1]^首次报道了该类型肺癌后，其逐渐受到研究者关注和认识^[2⇓⇓⇓⇓⇓⇓⇓⇓-11]^，因常被考虑为感染或炎症^[12]^，除少部分临床表现为呼吸系统常见症状外^[13]^，通常只有在癌症进展时才引起关注，这导致存在漏诊或误诊的风险^[14,15]^，荷兰-比利时肺癌筛查试验（Nederlands-Leuvens Longkanker Screenings Onderzoek, NELSON）发现22.7%的LCCA缺失或其鉴定延迟^[16]^。而且，部分小病灶或囊壁的增厚部分取材困难，早期难以通过活检评估病情^[12,17]^，这给患者的预后带来不良影响。研究^[18]^发现LCCA患者病理分期多为I期，但较同分期的非LCCA预后差，且LCCA囊壁较厚是其预后不良的相关因素^[19,20]^。但目前肺癌的肿瘤原发灶-淋巴结-转移（tumor-node-metastasis, TNM）分期仅评估肿瘤最大径，而忽略与LCCA预后相关的囊壁厚度，故肺癌TNM分期并不能良好地预测LCCA的预后。本文将通过对LCCA的定义、病因与发病机制、影像学特征与诊断依据、组织学和病理学特征及预后等多方面的研究进展进行综述。

## 1 LCCA的定义及影像学分型

Farooqi等^[15]^将原发肺恶性肿瘤的内部或边缘存在囊腔的一类病变定义为“LCCA”。在LCCA的定义确立前，这类病变曾被称为：“表现为薄壁囊肿的支气管癌（carcinoma of the bronchus presenting as thin-walled cysts）”^[3]^、“伪装为薄壁囊肿的癌（carcinoma masquerading as a thin walled cyst）”^[4]^、“支气管癌和大疱性疾病（bronchogenic carcinoma and giant bullous disease）”^[5]^、“大疱性肺疾病相关肺癌（lung carcinoma associated with bullous lung disease）”^[7]^、“长期肺囊肿中产生的支气管肺泡癌（bronchioloalveolar carcinoma arising in longstanding lung cysts）”^[8]^等。

与LCCA相关的囊腔样病变在计算机断层扫描（computed tomography, CT）影像中曾被称为“囊肿（cyst）”“大疱（bullae）”“气泡（bleb）”“囊腔”等，但未形成统一意见。根据Fleischner协会胸部影像术语的定义^[21]^，认为“肺气瘤（pneumatocele）”的描述更恰当，肺气瘤影像学上表现为肺内一个近似圆形、薄壁的空腔，可能由肺实质坏死和“止回阀”（check valve）造成的气道阻塞形成。影像学表现为肺内囊腔的病变可出现于LCCA中，也出现在良性病变中，如：肺结核、肺大疱、肺气肿、肺脓肿、支气管扩张等，且良性病变合并肺癌可能掩盖肺癌的诊断，故无论是恶性肿瘤继发生于囊腔还是恶性肿瘤导致囊腔的形成，与囊腔相关是LCCA的重要特征。

现已报道4种LCCA的主要影像学分类系统。Maki和Macalchi将LCCA根据影像学分为4型——I型：外生结节型；II型：内生结节型；III型：软组织延囊壁弥散增厚型；IV型：实性或非实性组织散布于多囊腔内^[22]^。Fintelmann在上述分类的基础上，开发了基于囊腔形态、病变的密度、囊腔囊数的新分类系统。LCCA分为I型：薄壁型；II型：厚壁型；III型：壁结节型；IV型：混合型4种类型（图1）。Zhu等^[23]^根据Jung^[60]^对LCCA动态演变不同阶段的特征获得在预测囊腔型肺腺癌（lung adenocarcinoma associated with cystic airspaces, LACA）病理浸润性更具临床价值的分类方式：I型：囊腔大小<6.5 mm的纯磨玻璃密度影（ground-glass opacity, GGO）；II型：囊腔大小≥6.5 mm的纯GGO；III型：囊腔伴部分实性结节；IV型：囊腔伴实性结节。其中Shen^[50]^和Zhu^[23]^采用的分型既描述了影像学特征，同时在预测患者病理是否为浸润性腺癌方面也具有重要意义。

**图1:**
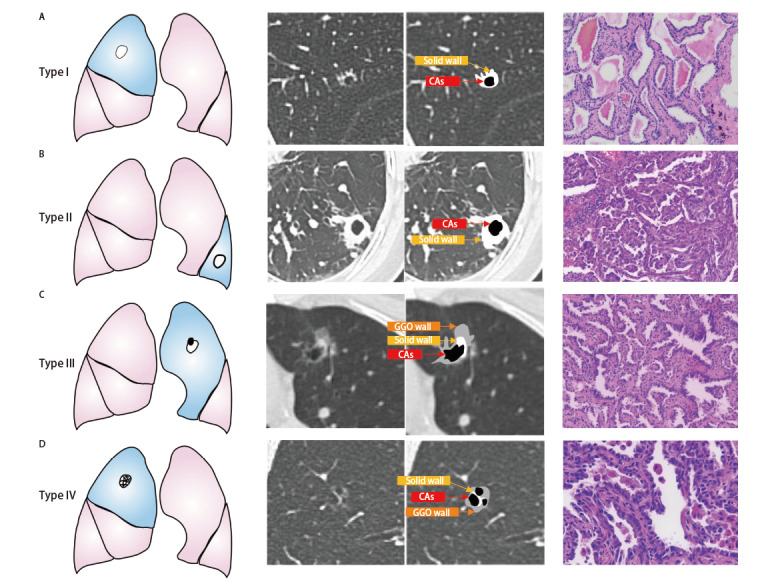
囊腔型肺癌的4种典型CT形态学分型。A：I型，平均壁厚<2 mm；B：II型，平均壁厚≥2 mm；C：III型，囊性空腔，伴有壁结节；D：IV型，组织混杂在囊性空腔内。4例患者术后病理均为腺癌（HE染色，×100）。

## 2 病因及发病机制

### 2.1 烟草暴露

LCCA的发病与吸烟存在一定的相关性^[5,6,11,19,22,24⇓⇓⇓⇓⇓⇓⇓⇓-33]^。Goldstein等^[5]^发现40-59岁的支气管肺癌患者的肺大疱发病率比无癌患者高6倍以上，大疱可能改变了段支气管顺应性，使得吸入的香烟烟雾等微粒沉积于肺泡上皮，导致细胞异型性产生和癌症的发生。Stoloff等^[6]^发现肺大疱患者患肺癌的相对危险度（relative risk, RR）为32，且Iwama等^[34]^认为LCCA患者可能对吸烟引起的DNA损伤更加敏感，故研究者^[6,11]^提出“潜在因素假说”——烟草损害了肺部的清除率，含有致癌物质，同时破坏肺组织间隔导致肺大疱的形成。但在无吸烟史的患者中也有LCCA的发生^[8,18,35⇓⇓-38]^，其中组织学类型为LACA在无吸烟史的患者中更常见^[39]^。

### 2.2 慢性阻塞性肺疾病（chronic obstructive pulmonary diseases, COPD）

LCCA的发生可能与COPD相关。Guo等^[20]^认为COPD患者慢性咳嗽、排痰困难导致的肺组织强度降低可能促进LCCA的产生。Araki等^[40]^发现肺气肿并发LCCA的可能性明显高于其他良性肺囊性疾病，Sheard等^[12]^也发现70.8%-85%的LCCA患者同时患有肺气肿，这提示肺气肿可能是LCCA的独立危险因素，Fintelmann等^[30]^也证实了LCCA的发生与肺气肿相关。

### 2.3 感染及瘢痕

LCCA的发生与伤口愈合、慢性纤维化存在一定联系，且瘢痕癌与LCCA相关^[31,41]^。Prichard等^[8]^认为无吸烟史的LCCA患者可能因囊腔内反复的炎症和瘢痕形成导致了LCCA的发生。Kaneda等^[19]^也认为空腔内的空气流量受限，微生物在大疱壁上的沉积，造成反复感染形成瘢痕与LCCA发生存在一定相关性。有研究^[42]^发现LCCA的囊腔内无液化坏死，提示囊腔形成可能与坏死无关。

### 2.4 多因素相互作用

LCCA的形成并非单一因素，而是多种机制联合作用的结果：在预先存在的囊腔中，气流受限导致的微生物沉积与反复囊腔感染出现的纤维性瘢痕，可能引起了烟草等致癌物的积累和感染囊腔上皮化生，导致囊腔癌变^[31,43]^。但仍不能确定LCCA囊腔与肺癌发生的先后关系，目前仅通过影像学表现把两种不同发病机制的疾病归类为LCCA^[39]^，未来还需要更多研究揭示LCCA的具体发病机制。

### 2.5 “止回阀”形成

LCCA中存在细支气管梗阻以及内衬于囊腔壁的肿瘤细胞^[29]^，而肿瘤细胞周边存在纤维组织增生^[44]^，两者造成气道的局灶性狭窄导致气体只能单向进入囊腔而难以排出^[45]^，形成“止回阀”。目前有学者^[35]^认为：“止回阀”的形成是导致LCCA的重要原因，其造成肺泡部分充气或含气量增加，形成非实性的肺癌结节。另有学者^[46]^认为：肿瘤的生长导致肺泡腔的脆性增加，并且肿瘤组织向内牵拉，也在“止回阀”的基础上促进了囊腔的形成。

## 3 LCCA的影像学特征与早期诊断

胸部CT是肺癌筛查和诊断的常用手段，通过分析LCCA的影像学特征对早期诊断LCCA具有重要意义^[12,47,48]^。LCCA可发生于任何肺叶，且多位于肺外周^[15,18,20,22,32,39,45,49]^。通过CT随访肺囊腔样病变的患者时，当囊腔出现囊壁增厚、囊壁上结节增大、新发结节或壁结节密度增加，考虑为LCCA的可能性增大^[22,50]^。对囊腔边缘不规则，尤其是随访后出现空洞变大^[20,36]^、胸膜凹陷征^[32]^、沿囊壁的壁结节、偏心结节或磨玻璃改变^[25,50]^、囊腔分隔、粗糙的蜂窝状囊壁的囊腔样病变也应该警惕为LCCA^[45,50]^。Mendoza等^[18]^的一项meta分析发现当病变存在囊腔及实性或磨玻璃结节成分时，就应怀疑为LCCA，影像学检查若发现囊壁进行性增厚和/或伴壁结节的囊腔样改变也应怀疑为LCCA^[12,18,30]^。目前研究发现除上述典型的LCCA影像学特征表现外，部分LCCA同样存在分叶征、毛刺征、胸膜凹陷征、血管穿行征等外周型肺癌的恶性征象^[13,17,51,52]^，且以血管穿行征及分叶征多见^[53]^，并与胸膜关系密切^[54]^。与空洞性肺结核相比，LCCA囊壁较薄，囊腔体积较大^[55]^，通过联合病灶最长径、囊腔最长径、囊壁最大厚度分析对鉴别肺结核与LCCA更具有价值^[56]^。另外，Shen等^[43]^发现LCCA的影像学病灶大小与病理肿瘤大小差异明显，尤其是在壁结节型（Shen-III型）和混合型（Shen-IV型）中。

常见的肺癌影像学表现的改变包括：肿瘤大小增加和减少，从磨玻璃到部分实性，部分实性到实性成分^[57⇓-59]^，LCCA的影像学表现也发生类似改变（图2）：有报道^[14]^称部分LCCA患者初诊为磨玻璃结节、亚实性结节和实性结节，在随访期间逐渐转变为囊腔样改变。Fintelmann等^[30]^也发现部分病例的结节出现早于囊腔，且这些囊腔常发生于结节旁无病变的正常肺组织；LCCA患者在CT随访时可能出现结节增大增多或囊壁增厚，这些改变可能导致LCCA完全转变为实性病灶^[18]^。Farooqi、Mascalchi和Tan等^[15,22,45]^也报道了随访的LCCA患者可发生囊壁增厚并转变为壁结节，或囊腔被实性组织占据进而转变为实性病灶（占比为75%-100%）。囊腔的形态改变也可能导致不同影像学分型之间的互相演变。Mascalchi等^[22]^在连续的CT复查中发现了多种影像学分型演变，如I型患者出现壁结节增大、全囊壁增厚演变为III型，III型患者囊壁上出现了外生性结节演变为I型，II型患者出现实性成分增加演变为IV型等。Fintelmann等^[30]^还观察到单囊腔型与多囊腔型LCCA的相互转变。Shen等^[50]^也报道了Shen-I型患者在随访期间出现囊壁新发结节并增大变为Shen-III型，或囊壁增厚转变为Shen-II型。上述研究提示LCCA可能是肺癌发展中的一种过程性病变。Jung等^[60]^通过27例LCCA患者的CT随访情况，总结了LCCA发展的4个阶段：（1）囊腔出现在非实性结节中间；（2）囊腔增大；（3）囊腔边缘出现实性成分；（4）实性成分逐步包绕囊腔并且增厚，囊腔逐渐变小。LCCA的演变进展模式反映了该类疾病的自然病程，同时该动态变化特征也为LCCA的诊断提供了一定参考依据。但正电子发射计算机断层显像（positron emission tomography/computed tomography, PET/CT）对LCCA患者的早期诊断尚存在局限性。有研究^[22,30,60⇓-62]^报道在行PET/CT检查的LCCA患者中，11.8%（2/17）的病灶无^18^F-脱氧葡萄糖（fluorodeoxyglucose, FDG）摄取，但术后病理为腺癌，其影像学分型多为Macalchi-I型和II型，而最大标准摄取值（maximum standardized uptake value, SUVmax）>5的病例多为Macalchi-III型和IV型，且23.5%（4/17）的SUVmax随时间逐渐增加。Fintelmann等^[30]^研究发现在LCCA患者中PET/CT检查结果阳性常与壁结节或囊壁相关，且认为当病灶的实性成分>8 mm时，PET/CT具有诊断价值。Toyokawa等^[63]^发现LACA患者PET/CT的SUVmax显著高于非囊腔型。因LCCA在PET/CT的SUV受多种因素影响，目前无大样本研究，故PET/CT在LCCA的诊断中的价值仍不确定。

**图2:**
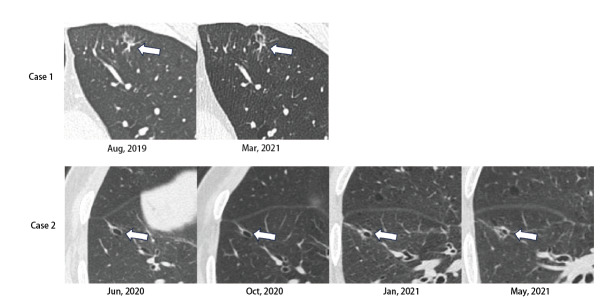
疾病进展的两种模式。病例1显示在随访过程中囊腔体积增大，实性成分增多。病例2表现为实性成分增多，分隔为多个囊腔。

活检困难导致了LCCA早期诊断困难，而通过胸部CT动态随访观察囊腔大小、囊壁厚度、新发结节和成分的改变可发现LCCA，但也增加了由长期随访所致的病情进展风险，且部分肺囊性病变患者因缺少胸部CT检查资料导致随访难度进一步提升。故评估LCCA的影像学特征，不仅对提高其早期诊断的准确率具有重要价值，也有利于通过影像学特征与临床特征之间的联系从而揭示其病因和发生机制。

## 4 LCCA的病理及组织学特征

CT影像学LCCA的主要组织学类型是腺癌^[49]^，且LACA中原位腺癌占比较少^[18,20,22,25,30⇓-32,36,39,45,46,50,64]^。与囊腔型肺鳞状细胞癌（lung squamous cell carcinoma associated with cystic airspaces, LSCA）相比，LACA的肿瘤实性成分占比更高，囊壁更厚^[39,63]^，且囊腔CT表现为薄壁、内外边缘光滑、磨玻璃征的LCCA多为原位腺癌或微浸润腺癌^[39,65]^，而浸润性LACA多表现为多发囊腔、不规则囊腔、胸膜凹陷征、分叶征及肺气肿等特点，且多为Shen-III型和IV型^[23]^。囊腔形态与病理亚型相关，且分化较差，其中薄壁型（囊腔壁厚度≤4 mm）LACA可能与侵袭性较低的病理亚型相关，而厚壁型（囊腔壁厚度>4 mm）的LACA患者的病理亚型以实体型为主^[29]^。Jung等^[60]^的研究结果发现，在LCCA中随着囊腔的扩大或囊腔壁成分由非实性转变为实性成分，LACA的病理亚型中贴壁型和腺泡型的比例由80%减少至42%，而实体型和微乳头型的比例由1.3%增加至33.6%。

LCCA的影像学表现与病理特征存在相关性。Byrne、Wu和Tan等^[14,42,45]^发现：囊壁不均匀是肿瘤细胞浸润程度和位置所致，不规则的囊腔边缘是由肿瘤细胞产生的纤维组织所致，囊腔内分隔由肿瘤细胞、支气管或血管产生的纤维组织组成。厚壁型LACA患者的血管浸润（以大血管浸润为主）、淋巴浸润、坏死、阻塞性肺炎、腔内脓肿、细支气管梗阻的发生率都显著高于薄壁型^[29,32,63]^。Jung等^[60]^发现在早中阶段时为纤维化薄壁衬于囊腔的边界，中晚阶段纤维化的囊壁更厚且被腺癌细胞覆盖，晚阶段可观察到浸润性腺癌细胞浸润纤维化区域，且晚阶段的LCCA患者易出现更晚的TNM分期及气腔内播散（spread through air spaces, STAS）。Shen等^[43]^发现对于呈现磨玻璃样浑浊的LCCA，肿瘤浸润性与肿瘤大小、实性成分、不包括囊腔的肿瘤体积及最大CT值相关，但亦有研究者^[23]^认为除多发囊腔、不规则囊腔形态和密度外，整个包括囊腔和病变成分的大小与LACA的浸润性密切相关。Toyokawa等^[63]^发现直径≤3 cm的LACA患者较非LACA患者病理分期较晚，其中，II期占6/18，III期占7/18，其主要原因是LACA更易侵犯胸膜、脉管；但也有研究者^[32]^发现较非LCCA，LCCA的病理分期暂无明显差异。Ma等^[39]^发现影像学表现为壁结节型的LCCA与较差的分化程度相关，且LSCA的分化较差。故LCCA病理组织学特征与囊腔形态和成分存在一定相关性，通过分析LCCA的影像学特征可预测其分化程度及是否为浸润性癌，进而指导LCCA治疗方案的选择。

目前，基于影像学特征及病理特征相关性研究，针对LCCA的治疗方式选择也取得了一定进展。Ma等^[39]^认为：对伴有囊腔的小纯磨玻璃结节，若CT表现为薄壁、内表面和边缘光滑，可根据术中冰冻切片进行亚肺叶切除；Shen-I型LACA患者可选择亚肺叶切除术；对于具有囊腔的鳞状细胞癌在出现薄壁型、壁结节型、囊腔较大、囊壁厚、实性壁成分时，建议首选肺叶切除术。但Toyokawa等^[63]^认为对早期LACA应采用肺叶切除术而非亚肺叶切除术。对巨大肺大疱行肺减容手术可改善患者肺功能，同时也能早期切除无法发现和诊断的LCCA^[10]^。

LCCA的病理特征与其影像学特征存在一定联系，通过影像学表现可预测其病理表现及浸润程度，LCCA的分期方式是否应区别于一般类型肺癌暂无明确结论，LCCA肿瘤细胞的分布方式、囊腔大小是否应该纳入肿瘤大小的测量仍需大样本量的研究来提供参考。

## 5 预后

一项meta分析^[18]^发现LCCA患者病理分期多为I期，但预后差，且与囊壁厚度存在相关性^[19,20]^。Watanabe等^[29]^厚壁组LACA患者的术后复发和远处转移率明显高于薄壁组，Ma等^[39]^也发现LCCA患者的中薄壁型（Shen-I型）预后较好，厚壁型（Shen-III型）预后最差。另外，Jung等^[60]^发现LACA患者影像学进展阶段与预后相关，后3个阶段的5年无复发生存（recurrence-free survival, RFS）率分别为100%、56%和16%，5年总生存（overall survival, OS）率分别为100%、70%和48%，复发率也逐渐升高，实性囊壁越厚，复发率越高，且亚实性结节的浸润性成分的体积大小比总体积大小的预测效果更好。Toyokawa等^[63]^研究认为LACA的无病生存期（disease-free survival, DFS）明显短于非LCCA，但OS无显著差异。

目前普遍认为：同体积大小的LCCA比一般类型肺癌的预后差，故同体积大小的病灶是否应区别于一般类型肺癌的管理模式以及对囊腔型相关肺结节的管理是否应该以病理为浸润前或早期浸润性腺癌的亚实性结节的方式监测或手术治疗仍需要大样本数据进一步完善^[16,66,67]^。

## 6 未来与展望

目前，LCCA对比非囊腔型肺癌的预后总体较差，但针对LCCA的管理方案尚未形成规范。现阶段对其影像学特征的研究使得对其早期诊断率有所提升，但仍需要多中心、大样本量研究数据以构建更为准确的LCCA早期诊断影像预测模型；对LCCA的分期方式目前暂无相关报道，仍使用世界卫生组织的肺癌TNM进行分期，但其病理特征与影像学特征的相关性和特殊性导致的该类患者预后较差，故是否需要新的分期方式对LCCA进行管理有待更多研究佐证。影响LCCA预后的因素涉及患者、肿瘤及治疗方法等多个方面，现在人工智能（artificial intelligence, AI）的发展可能带来影像学对肺癌诊断的突破，未来需要大样本、多中心调查来揭示流行病学特点，并完善影像学、病理学、分子分型与驱动基因突变等多维度的相关性研究，为LCCA诊疗策略的确立提供证据支持，从而提升LCCA的治疗水平，改善患者预后。

**Competing interests**

The authors declare that they have no competing interests.
